# A review of the Indonesian species of the family Signiphoridae (Hymenoptera, Chalcidoidea), with description of three new species

**DOI:** 10.3897/zookeys.897.38148

**Published:** 2019-12-09

**Authors:** Stefan Schmidt, Hasmiandy Hamid, Rosichon Ubaidillah, Samantha Ward, Andrew Polaszek

**Affiliations:** 1 SNSB-Zoologische Staatssammlung München (ZSM), Münchhausenstr. 21, 81247 Munich, Germany Zoologische Staatssammlung München Munich Germany; 2 Fakulty of Agriculture, Andalas University, Padang 25163, Sumatera, Indonesia Andalas University Padang Indonesia; 3 Zoology Division, Museum Zoologicum Bogoriense (MZB), Research Center for Biology, Indonesian Institute of Sciences, Jl. Raya Jakarta-Bogor KM 46, Cibinong, Bogor 16911, Java, Indonesia Research Center for Biology, Indonesian Institute of Sciences Cibinong Indonesia; 4 School of Biosciences, The University of Melbourne, Bio21 Institute, 30 Flemington Road, Parkville VIC 3052, Australia The University of Melbourne Parkville Australia; 5 Department of Life Sciences, Natural History Museum, London, UK Department of Life Sciences, Natural History Museum London United Kingdom

**Keywords:** chalcid wasps, hyperparasitoids, parasitoids, south-east Asia, taxonomy

## Abstract

The Indonesian species of the family Signiphoridae (Hymenoptera, Chalcidoidea) are revised. Three species of *Chartocerus* are described as new (*Chartocerus kartiniae* Polaszek & Schmidt, **sp. nov.**, *C. sumatrensis* Schmidt & Polaszek, **sp. nov.**, and *C. javensis* Schmidt & Ubaidillah, **sp. nov.**) and four species of *Signiphora*, viz., *S. bennetti* Woolley & Dal Molin, *S. flavella* Girault, *S. perpauca* Girault and *S. bifasciata* Ashmead, are diagnosed.

## Introduction

The Signiphoridae are a small family of Chalcidoidea with currently 88 described species in four genera (Noyes 2019). The family has a worldwide distribution, with the majority of species occurring in the tropics. Signiphorids are known primarily as hyperparasitoids of scale insects, mealybugs, and whiteflies (Hemiptera, Sternorrhyncha) through encyrtid or aphelinid primary parasitoids, while some are obligate primary parasitoids on these sternorrhynchan hosts. Species of *Clytina* Erdös and most of the *Signiphora dipterophaga* species group are primary parasitoids of Diptera puparia (Woolley 1988).

Species of the family Signiphoridae are distinguished from other families of chalcid wasps and can readily be recognised by the following combination of characters: (1) propodeum with a characteristic large triangular median area, (2) antenna with 1 to 4 short, often ring-like flagellomeres, and a long, undivided clava, (3) metasoma broadly attached to the mesosoma, and (4) wings only with marginal setae, the disc bare, occasionally with one large discal seta.

The present revision includes seven species of the family Signiphoridae from Indonesia, three of them described here as new to science, in two genera. The specimens were obtained as part of several capacity building and biodiversity research projects in Indonesia, in particular the IndoBiosSys project that aimed, amongst other goals, at creating inventories of the Indonesian entomofauna using DNA barcoding. Here we present the results for a family of chalcid wasps, the Signiphoridae, that was recorded only recently for the first time in Indonesia, with a single species, *Signiphora bifasciata* Ashmead, in the Bogor Botanic Garden as a parasitoid of the introduced cycad aulacaspis scale (*Aulacaspis yasumatsui* Takagi (Hemiptera, Diaspididae) (Muniappan et al. 2012).

## Materials and methods

The specimens were collected during field training courses at the universities in Padang, Sumatra (Andalas University) and Malang, Java (Brawijaya University), as part of capacity building activities for young Indonesian entomologists, and as part of the Indonesian Biodiversity Discovery Project (Cancian de Araujo et al. 2017). The National Park has been recognised as one of the largest remaining tropical rain-forest ecosystems in Java, being designated as a National Park in 2003 with a present area of about 113,357 hectares. During the IndoBioSys Project, Malaise traps and Yellow Pan traps were employed in the Mount Halimun-Salak National Park in West Java. In addition, chalcid wasps were collected using a screen-sweep net (cf. Noyes 1982).

The descriptions of the three new species are based on specimens that were borrowed from the Museum Zoologicum Bogoriense, Research Center for Biology, Indonesian Institute of Sciences. Data on genetic material contained in this paper and the Barcode of Life Database (BOLD) are published for non-commercial use only, according to the agreements with the providing country of the analysed samples. Use by third parties for purposes other than non-commercial scientific research may infringe the conditions under which the genetic resources were originally accessed and should not be undertaken without obtaining consent from the corresponding author of the paper and/or obtaining permission from the original providers of the genetic material.

### DNA sequencing

For DNA studies, whole specimens were sent to the Canadian Centre for DNA Barcoding (CCDB) in Guelph, Canada, for DNA extraction and barcode sequencing, and subsequent recovery of vouchers for preparation and morphological study. DNA extraction, PCR amplification, and sequencing were conducted at the CCDB using standardised high-throughput protocols (Ivanova et al. 2006, deWaard et al. 2008, http://www.ibolproject.org/resources.php). The 658bp target region, starting from the 5’ end of the mitochondrial cytochrome c oxidase I (COI) gene, includes the DNA barcode region of the animal kingdom (Hebert et al. 2003). All specimen data are accessible on BOLD through the following doi: dx.doi.org/10.5883/DS-INDOSIG. The specimen data include collecting locality, geographic coordinates, elevation, collector, one or more digital images, identifier, and voucher depository. Sequence data can be obtained through BOLD and include a detailed LIMS report, primer information, and access to trace files. These data are also available through GenBank (Accession nos MH407234–MH407243).

### Data analysis

Sequence divergence statistics were calculated using the Kimura two parameter model of sequence evolution (Kimura 1980), as it is commonly applied in the analysis of DNA barcode sequence data because it evaluates the substitution type (i.e., transitions vs transversions) in addition to the number of nucleotide substitutions between sequences. Barcode Index Numbers (BINs) were assigned by the BOLD system, representing globally unique identifiers for clusters of sequences that correspond closely to biological species (Ratnasingham and Hebert 2013). For BIN assignment, a minimum sequence length of 500 bp is required, and sequences between 300 and 500 bp can join an existing BIN but will not create or split BINs. Sequences were aligned using the BOLD Aligner (amino acid-based hidden Markov models). The analyses are based on sequences with a minimum length of 500 bp and <1% ambiguous bases. Genetic distances and summary statistics were calculated using analytical tools in BOLD and are given as mean and maximum pairwise distances for intraspecific variation and as minimum pairwise distances for interspecific variations.

### Morphology and imaging

Morphological terminology and the format for species descriptions follow Hayat (2009). Photographs were made using a Leica Ortholux compound microscope with Nomarski Differential Interference Contrast (DIC) illumination and a Leica DM 5000 B with DIC. Images were processed using the stacking software AutoMontage (Synoptics, Cambridge, UK) and Helicon Focus (version 7.0), and further edited using Adobe Photoshop CC 2019. Plates were compiled with Adobe Illustrator CS6.

### Acronyms of depositories

**MZB**Museum Zoologicum Bogoriense (MZB), Research Center for Biology, Indonesian Institute of Sciences, Cibinong, Indonesia.

**NHMUK** Natural History Museum, London, UK

**ZSM** SNSB – Zoologische Staatssammlung München, Munich, Germany

## Taxonomy

### Chartocerus

Taxon classificationAnimaliaHymenopteraSigniphoridae

Motschulsky, 1859

084186DF-1751-5F54-9861-D4340B71F543

#### Diagnosis.

Body dark brown or black, often with metallic luster. Fore tibial spur simple, without comb of setae (cf. Fig. 3H). Propodeum posteriorly without lamelliform process (except in *C. kartiniae* with a suggestion of a lamelliform process on the median sclerite). Female antenna with 4 anelli (except in *C. kartiniae* with 2 anelli), male with 3 anelli. Seta M6 on fore wing marginal vein present, and with additional seta between M2 and M3 (M2b, Fig. 4E). Occipital margin rounded or acute. Mesofemur with 3 or 4 long spines (cf. Fig. 4D).

### Chartocerus javensis

Taxon classificationAnimaliaHymenopteraSigniphoridae

Schmidt & Ubaidillah sp. nov.

1047AE77-C885-5F23-8142-C73FD5185B74

http://zoobank.org/4BBC4A9C-59D1-4AF0-996D-09931C0F836E

Figs 1A–H , 2A–G

#### Material examined.

***Holotype.*** Indonesia • ♀ (on slide); East Java, Jalan Kandangan, Kasembon; 7.808S, 112.313E; 305 m a.s.l.; 14-Jul-2012; S. Schmidt leg.; screen-sweep net; MZB; specimen ID: BC-ZSM-HYM-20770-C04. ***Paratype***. Indonesia • ♂ (on slide); East Java, Jalan Kandangan, Kasembon; 7.808S, 112.313E; 305 m a.s.l.; 14-Jul-2012; S. Schmidt leg.; screen-sweep net; MZB; specimen ID: BC-ZSM-HYM-20770-C11.

#### Diagnosis.

**Figure 1.**
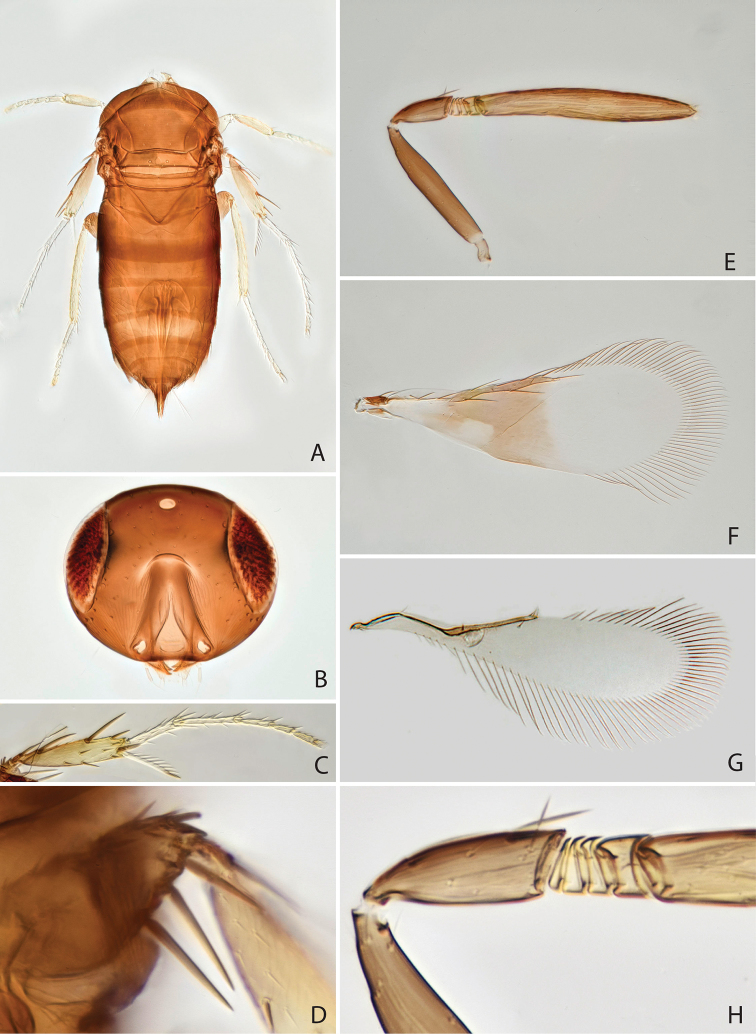
*Chartocerus javensis* Schmidt & Ubaidillah, sp. nov., female holotype **A** body in dorsal view **B** head in frontal view **C** middle leg **D** apex of middle femur **E** antenna **F** fore wing **G** hind wing **H** pedicel and flagellum base.

Head and body dark brown (Fig. 1A, B), fore wing behind marginal vein with infuscation except hyaline area near posterior margin (Fig. 1F). Antenna (in female) with 4 anelli (Fig. 1H). Clava elongate, about 6.7 times as long as broad and 1.5 times as long as scape length (Fig. 1E). Fore wing marginal fringe slightly longer than half the length of the wing disc (Fig. 1F). Midtibial spur subequal in length to corresponding basitarsus (Fig. 1C).

#### Description (female holotype).

***Colour.*** Head and body dark brown (Fig. 1A, B), legs brown with tarsi pale (Fig. 1A), antenna brown (Fig. 1E). Fore wing with brown infuscation behind marginal vein, leaving a hyaline area posteriorly in basal half of the infuscation (Fig. 1F), and a brown longitudinal stripe in basal cell.

***Morphology.*** Head 1.78 times as broad as frontovertex width (Fig. 1B), frontovertex width slightly less than length of clava (0.84), distance from mouth margin to facial ridge 0.61 times head height. Antenna with 4 anelli, F4 longer than each of the preceding segments, clava 6.7 times as long as broad and 1.51 times as long as scape length, pedicel 0.45 times as long as scape length (Fig. 1E). Midlobe of mesoscutum with 4 setae in anterior half and 6 setae in posterior half, scutellum with 7 setae along posterior margin. Fore wing 2.7 times as long as broad (Fig. 1F), longest setae of marginal fringe 0.56 times as long as width of disc, seta M1 absent, M2b present. Mesofemur ventrally with 3 long spines (Fig. 4D). Midtibial spur subequal in length to corresponding basitarsus (Fig. 1C), the latter 0.55 times as long as midtibia. Ovipositor nearly twice as long as midtibia (1.82) and 1.29 times as long as hind tibia.

#### Male.

**Figure 2.**
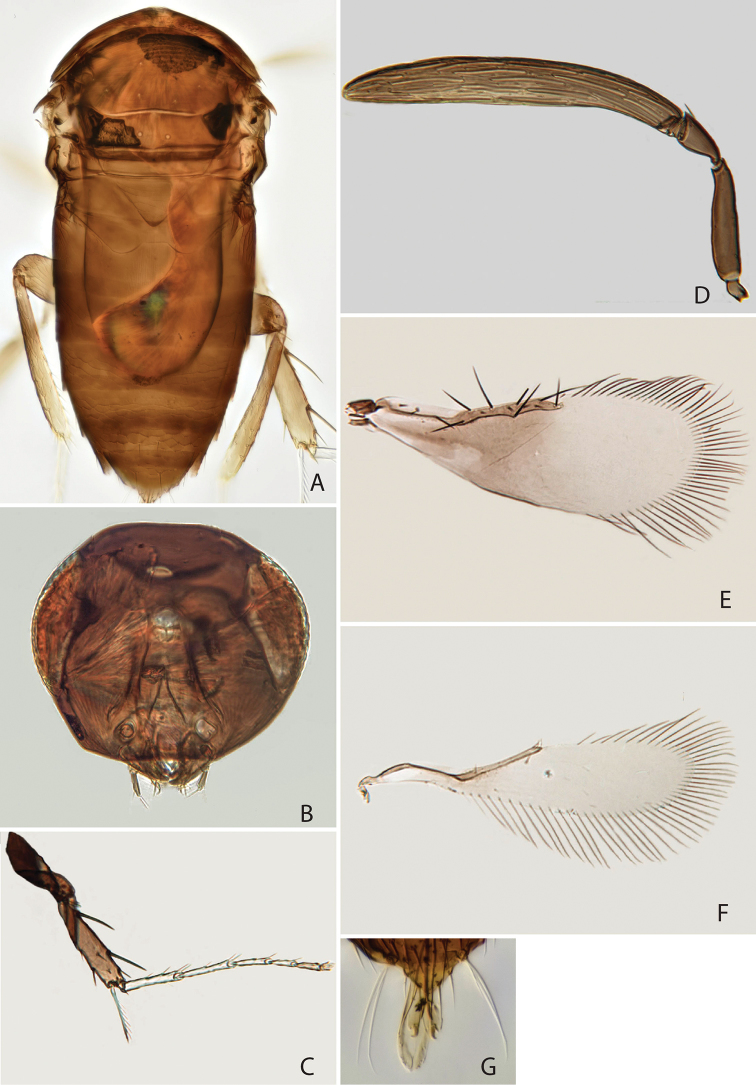
*Chartocerus javensis* Schmidt & Ubaidillah, sp. nov., male paratype **A** body in dorsal view **B** head in frontal view **C** middle leg **D** antenna **E** fore wing **F** hind wing **G** genitalia.

Colour and structure similar to female (Fig. 2A, B), but antenna (Fig. 2D) with 3 anelli and longer, clava 7.28 times as long as broad and 2.76 times as long as scape (Fig. 2D). Setae of fore wing (Fig. 2E) stouter than in female. Genitalia as in Fig. 2G, phallobase with a pair of setae, digitus about 3 times as long as broad, strongly curved at apex.

#### Host.

Unknown.

#### Distribution.

Only known from the type locality near Malang, East Java, Indonesia.

#### Etymology.

The species is named after the island of Java.

#### DNA barcode data.

The species exhibits an intraspecific variation of 0.2% and a distance of 12.2% to the nearest neighbour species, *C. sumatrensis* sp. nov. (Suppl. material 1, Genbank Accession Numbers: MH407241, MH407242).

#### Remarks.

The species is similar to *Chartocerus kerrichi* (Agarwal) and *C. sumatrensis* sp. nov. but can be distinguished from the former by the longer clava (6.7 times as long as broad, compared to 4.5 times in *kerrichi*), the longer fore wing marginal fringe (0.56 times as long as width of disc compared to 0.36–0.40 times in *kerrichi*), and the longer midtibial spur (subequal in length to corresponding basitarsus whereas distinctly shorter in *kerrichi*). From *C. sumatrensis* sp. nov. it can be separated by the shorter ovipositor (less than 2.0 times as long as midtibia, compared to 2.9 times in *sumatrensis*) and the longer midtibial spur (subequal in length to corresponding basitarsus, compared to 0.7 times in *sumatrensis*).

### Chartocerus kartiniae

Taxon classificationAnimaliaHymenopteraSigniphoridae

Polaszek & Schmidt sp. nov.

EE92951F-2478-5CD0-849F-C4FC76DB567A

http://zoobank.org/FB55530A-5082-40D9-81B8-795DB3F28C44

Fig. 3A–I

#### Material examined.

***Holotype.***Indonesia • ♀ (on slide); West Java, Mount Halimun-Salak National Park, waterfall; 6.71250S, 106.52305E; 1100 m a.s.l.; 18-Sept-2015; A. Polaszek leg.; screen-sweep net; MZB; specimens ID: DNA 1317.

#### Diagnosis.

**Figure 3.**
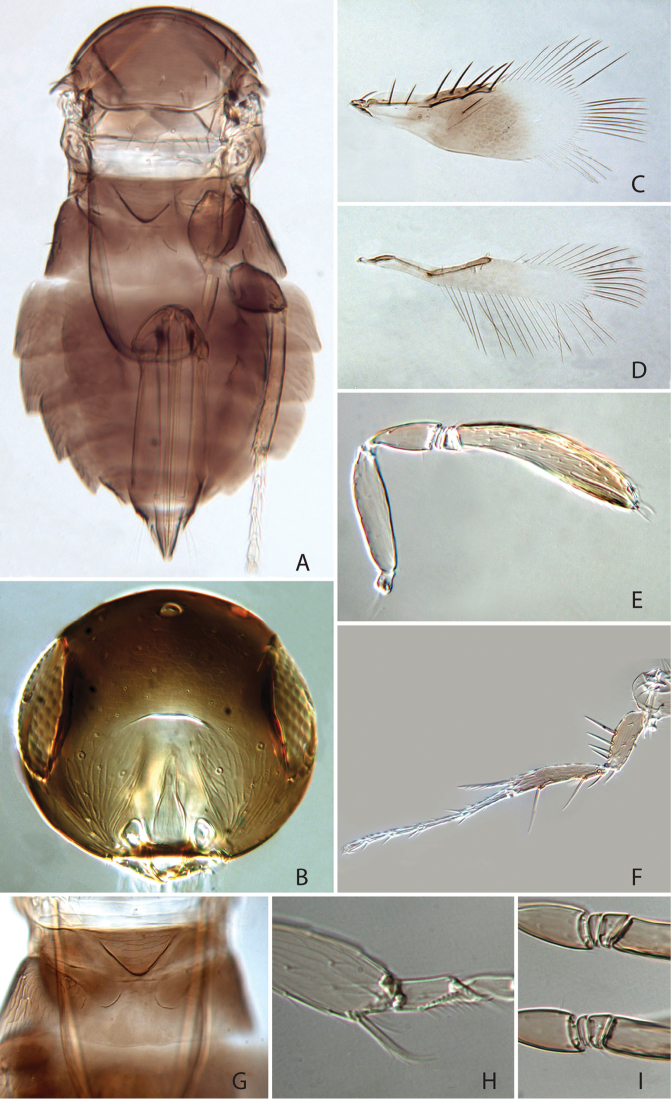
*Chartocerus kartiniae* Polaszek & Schmidt, sp. nov., female holotype **A** body in dorsal view **B** head in frontal view **C** fore wing **D** hind wing **E** antenna **F** middle leg **G** posterior mesosoma and anterior metasoma **H** apex of fore tibia with tibial spur **I** flagellum base.

*Chartocerus kartiniae* sp. nov. is unique among Signiphoridae and is provisionally placed in *Chartocerus* on a balance of genus-level characters. The female can be quickly diagnosed among all *Chartocerus* species by the following combination of characters: metanotum pale (Fig. 3A); fore wing with discal seta (Fig. 3C); antennal funicle 2-segmented (Fig. 3I).

#### Description (female holotype).

***Colour.*** Head and body dark brown (Fig. 3A, B), with metanotum distinctly pale in contrast, legs pale brown with fore tibiae and all tarsi pale yellow (Fig. 3F), antenna yellow-brown with radicle and scape paler (Fig. 3E). Fore wing with brown infuscation centrally and on marginal vein (Fig. 3C).

***Morphology.*** Head 1.52 times as broad as frontovertex width (Fig. 3B), frontovertex width 0.87 times length of clava. Antenna with only 5 antennomeres (excluding radicle); antenna with 2 anelli, clava 5.5 times as long as broad and 1.7 times as long as scape length, pedicel about 0.5 times as long as scape length (Fig. 3E). Midlobe of mesoscutum with 7 setae in posterior half, scutellum with 7 setae along posterior margin. Propodeum with a suggestion of a lamelliform process on the median sclerite. Fore wing 2.8 times as long as broad (Fig. 3C), longest setae of marginal fringe 0.78 times as long as width of disc, all marginal vein setae present, stigmal seta long; discal seta present. Foretibial spur bifid, without a comb (Fig. 3I). Midtibial spur 0.9 times as long as corresponding basitarsus (Fig. 3F), the latter 0.57 times as long as mid tibia. Mesofemur with four long spines anteroventrally. Ovipositor 2.0 times as long as mid tibia and 1.5 times as long as hind tibia.

#### Host.

Unknown.

#### Distribution.

Known only from the type locality at Mount Halimun, West Java, Indonesia.

#### Etymology.

The species is named after Raden Adjeng Kartini.

#### DNA barcode data.

No sequence data were available for analysis.

#### Remarks.

This species is unique for the genus, having only two funicle antennomeres, and a prominent discal seta on the fore wing. The pale metanotum is unusual (or possibly unique) among known species of *Chartocerus* (J.B. Woolley, pers. comm.). The foretibial spur is bifid and without a comb, supporting placement of the species in the genus *Chartocerus*. A full DNA barcode sequence could not be obtained for the single specimen, but analysis of an available DNA fragment suggests membership of the *Chartocerus* lineage (distant from *Signiphora* and *Thysanus* Walker), and a possible sister-group relationship to remaining *Chartocerus* for which DNA data are available.

### Chartocerus sumatrensis

Taxon classificationAnimaliaHymenopteraSigniphoridae

Schmidt & Polaszek sp. nov.

924AB8E8-964D-5109-ADFA-F59262AF6655

http://zoobank.org/D19FF62E-3F71-4DFB-A9B7-305C298CC31A

Fig. 4A–F

#### Material examined.

***Holotype.*** Indonesia • ♀ (on slide); West Sumatra, Padang, Universitas Andalas campus; 0.9043S, 100.4802E; 500 m a.s.l.; 17-Jun-2012; S. Schmidt leg.; screen-sweep net; MZB; specimens ID: BC-ZSM-HYM-05406-H10.

#### Diagnosis.

**Figure 4.**
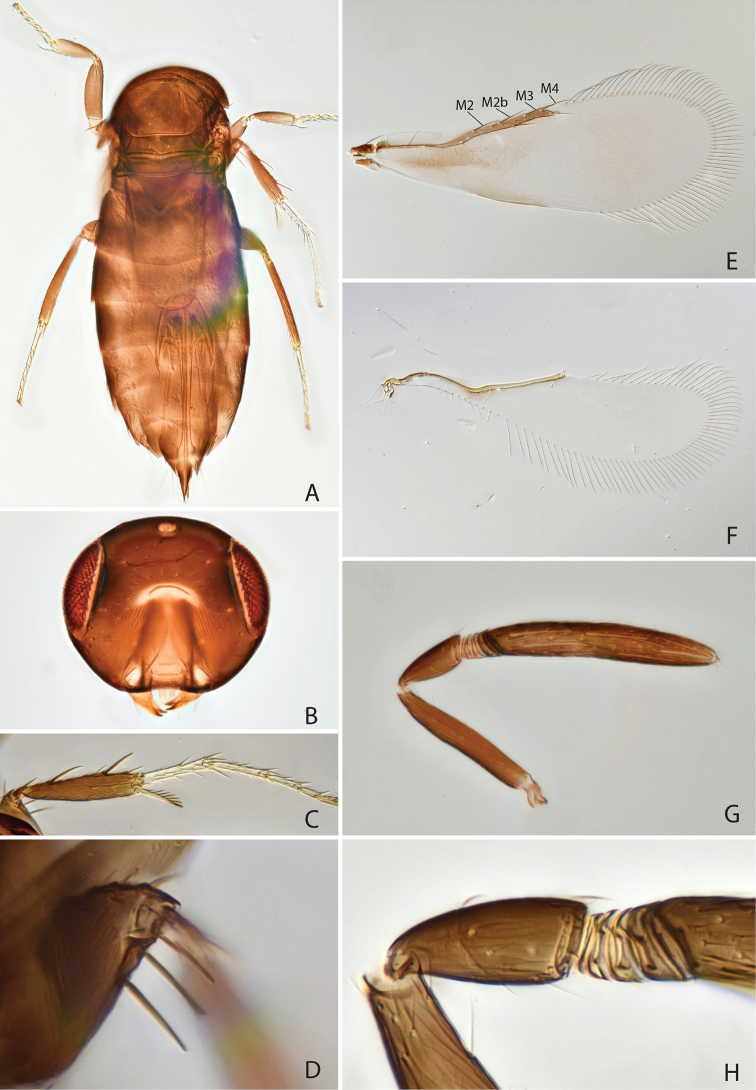
*Chartocerus sumatrensis* Schmidt & Polaszek, sp. nov., female holotype **A** body in dorsal view **B** head in frontal view **C** middle leg **D** apex of middle femur **E** fore wing **F** hind wing **G** antenna **H** pedicel and flagellum base.

Head and body dark brown (Fig. 4A, B), fore wing basally with brown infuscation (Fig. 4E). Antenna (in female) with 4 anelli (Fig. 4H). Clava elongate, about 6 times as long as broad and 1.5 times as long as scape length (Fig. 4G). Fore wing 2.9 times as long as broad, anteriorly with 4 setae, posterior margin of hind wing disc slightly rounded.

#### Description (female holotype).

***Colour.***Head dark brown (Fig. 4A, B), legs brown with tarsi pale yellow (Fig. 4C), antenna brown with radicle and basal 3 funicle antennomeres pale yellow (Fig. 4G). Fore wing basally with brown infuscation, and a darker patch behind distal part of submarginal vein and proximal part of marginal vein, and longitudinal dark patch along basal part of hind margin (Fig. 4E).

***Morphology.***Head 1.69 times as broad as frontovertex width (Fig. 4B), frontovertex width subequal to length of clava, distance from mouth margin to facial ridge 0.66. Antenna with 4 anelli, increasing in size towards apex (Fig. 4H), clava about 6 times as long as broad and 1.5 times as long as scape length, pedicel about 0.5 times as long as scape (Fig. 4G). Midlobe of mesoscutum with 11 setae in posterior half, scutellum with 8 setae along posterior margin. Fore wing 2.9 times as long as broad (Fig. 4E), longest setae of marginal fringe 0.38 times as long as width of disc, seta M1 absent, M2b present (Fig. 4E). Hind margin of hind wing disc slightly rounded and nearly parallel to anterior margin (Fig. 4F). Mesofemur ventrally with 3 long spines (Fig. 4D). Midtibial spur 0.7 times as long as corresponding basitarsus (Fig. 4C), the latter 0.53 times as long as midtibia. Ovipositor 2.9 times as long as midtibia and 2.0 times as long as hind tibia.

#### Host.

Unknown.

#### Distribution.

Only known from the type locality near Padang in West Sumatra.

#### Etymology.

The species is named after the island of Sumatra.

#### DNA barcode data.

A single, full-length barcode sequence of the species is separated from its nearest neighbour species, *C. javensis* sp. nov., by 12.2% (Suppl. material 1, Genbank Accession Number: MH407238).

#### Remarks.

The species is similar to *Chartocerus kerrichi* and *C. javensis* sp. nov., but can be distinguished from the former by the longer ovipositor (2.9 times as long as midtibia, compared to 2 times in *kerrichi*) and the more slender clava (about 6 times as long as broad, compared to 4.5 times in *kerrichi*). From *C. javensis* sp. nov. it can be separated by the longer ovipositor (2.9 times as long as midtibia, compared to less than 2 times in *javensis*) and the shorter midtibial spur (0.7 times as long as corresponding basitarsus, compared to subequal in *javensis*).

### Signiphora

Taxon classificationAnimaliaHymenopteraSigniphoridae

Ashmead, 1880

4740BF3C-A970-56E5-A348-02C7D16696B2

#### Diagnosis.

Colour variable, pale yellow to completely dark brown or black. Occipital margin acute. Antenna with 3, rarely 1–2 or 4 anelli. Mesoscutum from bare to setose with up to 100 setae. Propodeum posteriorly with lamelliform process. Fore tibial spur with a comb of setae. Fore wing submarginal vein with one or two setae, marginal vein dorsally with (4–)6 setae. Mesofemur with 1 or 2 bristles.

### Signiphora bennetti

Taxon classificationAnimaliaHymenopteraSigniphoridae

Woolley & Dal Molin

1039050E-25F5-5518-A845-E919838D7C52

Fig. 5A–F

Signiphora bennetti Woolley & Dal Molin, 2017: 19–23.

#### Material examined.

Indonesia • 4 ♀ (on slide); West Java, Mount Halimun-Salak National Park, waterfall; 6.71250S, 106.52305E; 1100 m a.s.l.; 18-Sept-2015; A. Polaszek leg.; Yellow Pan trap; MZB DNA 1321; ZSM DNA 1318; NHMUK DNA 1319-20.

#### Diagnosis (female).

**Figure 5.**
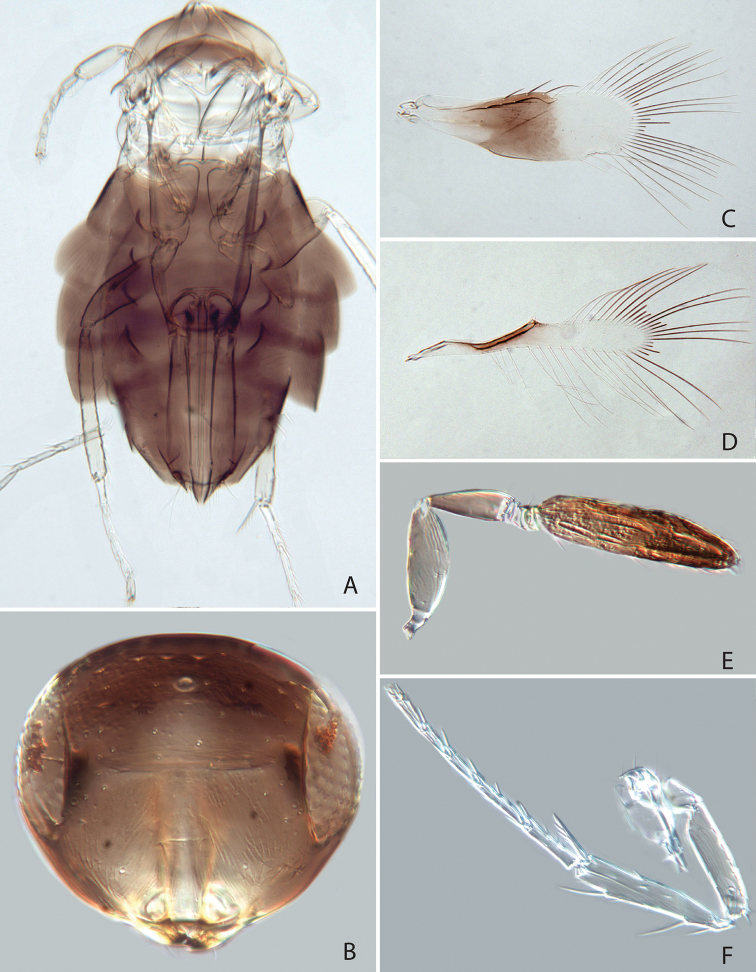
*Signiphora bennetti* Woolley & Dal Molin, female **A** body in dorsal view **B** head in frontal view **C** fore wing **D** hind wing **E** antenna **F** middle leg.

Female with pale band from distal mesoscutum to the proximal propodeal triangle. Antenna with 3 anelli (Fig. 5E). Marginal vein dorsally with 3 setae, seta M1 missing (Fig. 5C), usually with minute seta distal to M2.

**Description (female). *Colour***. Head and body dark brown (Fig. 5A, B), with distal half of mesoscutum, scutellum, metanotum and anterior part of propodeal triangle distinctly pale in contrast; legs pale with hind femora brown (Fig. 5A), antenna yellow-brown with clava darker, increasingly so distally (Fig. 5E). Fore wing with brown infuscation centrally and on marginal vein (Fig. 5C).

***Morphology.*** Head 1.5 times as broad as frontovertex width (Fig. 5B), frontovertex width 1.04 times length of clava. Antenna with 6 antennomeres and with 3 anelli, clava 4.6 times as long as broad and 1.7 times as long as scape length, pedicel 0.6 times as long as scape length (Fig. 5E). Midlobe of mesoscutum with 2 fine setae in posterior half, scutellum with 4 fine setae along posterior margin. Fore wing 3.5 times as long as broad (Fig. 5C), longest setae of marginal fringe 1.52 times as long as width of disc, marginal vein seta M1 absent, M5 absent in some paratypes; stigmal seta long (Fig. 5C), vestigial seta base/socket present between M5 and M6 on lower edge of marginal vein, discal seta absent (Fig. 5C). Midtibial spur 0.84 times as long as corresponding basitarsus (Fig. 5F), the latter 0.46 times as long as midtibia. Ovipositor 1.8 times as long as midtibia and 1.4 times as long as hind tibia.

#### Host.

A primary parasitoid, commonly associated with *Melanaspis smilacis* (Comstock) (Hemiptera, Diaspididae) in the New World, a species also widely distributed in SE Asia. Also recorded from *Hemiberlesia oxycoccus* (Woglum); *Melanaspis obscura* (Comstock); *Pseudaulacaspis pentagona* Targioni Tozzetti; *Comstockaspis perniciosa* (Comstock) and *Aspidiella sacchari* (Comstock) (Woolley and Dal Molin 2017).

#### Distribution.

Very widespread in the New World (Woolley and Dal Molin 2017).

#### DNA barcode data.

The species exhibits an intraspecific variation of 0.2% and a distance of 12.5% to the nearest neighbour species, *S. flavella* (Suppl. material 1, Genbank Accession Numbers: MN520843, MN520844).

#### Remarks.

The species belongs to the *Signiphora flavopalliata* Ashmead species group and can be separated from other species in the group by the central dorsal pale band extending from the distal mesoscutum to the proximal propodeal triangle (female), absence of M1 from the marginal vein, and the presence of a minute anterior seta on the marginal vein distal to M2 (fig. 38 in Woolley and Dal Molin 2017), though this seta is not present in every specimen.

### Signiphora flavella

Taxon classificationAnimaliaHymenopteraSigniphoridae

Girault

A32AECC2-910B-5D98-AE00-B3A538DC69AF

Fig. 6A–F

Signiphora flavella Girault, 1913: 214. Female. For a full list of synonyms see Woolley and Dal Molin (2017).

#### Material examined.

Indonesia: • 4 ♀ (on slide); East Java, Malang, Selorejo, Junrejo; 7.940S, 112.529E; 1015 m a.s.l.; 1-Jul-2012; S. Schmidt leg.; screen-sweep net; MZB BC-ZSM-HYM-20770-G02, BC-ZSM-HYM-20770-E12, BC-ZSM-HYM-20770-G04 (slide with right fore wing only), BC-ZSM-HYM-20770-F12 • 2 ♀; same data as preceding; ZSM BC-ZSM-HYM-20770-F05, BC-ZSM-HYM-20770-F09 • 1 ♀; same data as preceding; NHMUK BC-ZSM-HYM-20770-F10 • 2 ♀; same locality; 8-Jul-2012; S. Schmidt leg.; screen-sweep net; MZB • 1 ♀; East Java, Jalan Kandangan, Kasembon; 7.808S, 112.313E; 305 m a.s.l.; 14-Jul-2012; S. Schmidt leg.; screen-sweep net; NHMUK.

#### Diagnosis (female).

**Figure 6.**
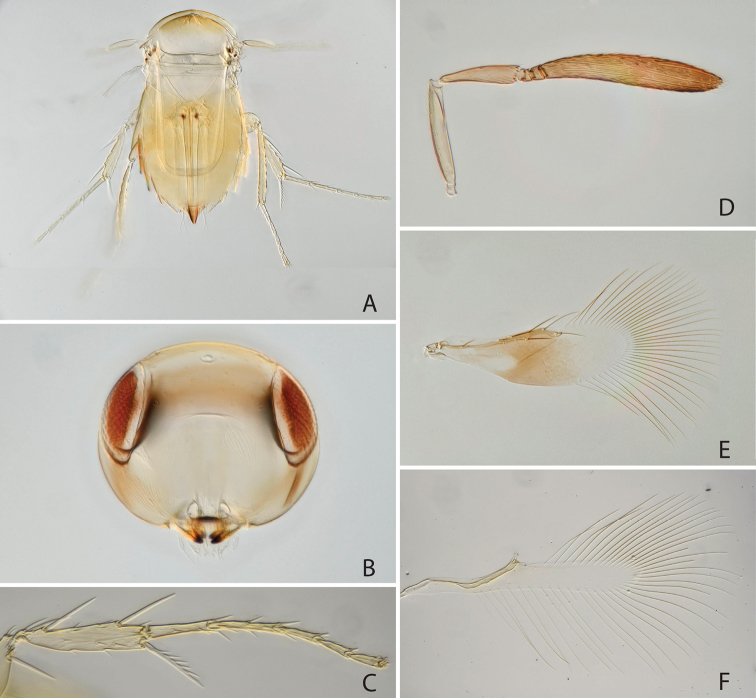
*Signiphora flavella* Girault, female **A** body in dorsal view **B** head in frontal view **C** middle leg **D** antenna **E** fore wing **F** hind wing.

Colour of body yellow (Fig. 6A) with variable extent of brown markings, clava only apically or completely dusky brown (Fig. 6D). Discal seta on fore wing missing, seta M1 on marginal vein present (rarely absent) (Fig. 6E). Mt8 in female transverse, without a medial emargination.

#### Description (female).

***Colour.*** Body yellow (Fig. 6A), head yellow except occiput with curved brown band along occipital carina (Fig. 6B). Antenna with scape yellow and flagellum brown, apical half of clava darker brown (Fig. 6D). Fore wing with dark band behind marginal vein that is proximally and distally diffusely delimited (Fig. 6E). Third valvula brown.

***Morphology.*** Head 1.76 times as broad as frontovertex width (Fig. 6B), frontovertex width 0.77 times length of clava, distance from mouth margin to facial ridge 0.57. Antenna with 3 anelli, F1 0.4 times as long as broad, F2 0.8 times as long as broad, F3 subquadrate, clava about 6 times as long as broad and 1.7 times as long as scape length (Fig. 6D), pedicel 0.77 times as long as scape length. Midlobe of mesoscutum with 2 setae on disc and anteriorly with 6 setae, scutellum with 6 setae along posterior margin. Fore wing 2.9 times as long as broad (Fig. 6E), longest setae of marginal fringe 1.4 times as long as width of disc, seta M1 present, M2b absent. Midtibial spur subequal in length to corresponding basitarsus (Fig. 6C), the latter 0.56 times as long as midtibia. Ovipositor 2.1 times as long as midtibia and 1.4 times as long as hind tibia.

#### Male.

The species reproduces primarily parthenogenetically; males are very rare (Woolley and Dal Molin 2017) and were not recorded in the study area.

#### Host.

Polyphagous on many species of Diaspididae (Hemiptera). For a full list of host records see Woolley and Dal Molin (2017).

#### Distribution.

Cosmopolitan and, apart from Indonesia, occurring in the following countries (after Woolley and Dal Molin 2017): Algeria, Argentina, Australia, Brazil, Chile, Honduras, Honduras, Greece, India, Israel, Mexico, Morocco, New Zealand, Peru, Puerto Rico, South Africa, Spain, Trinidad and Tobago, USA, Venezuela.

#### DNA barcode data.

The sequence data did not reveal any intraspecific variation and a distance of 9.7% to the nearest neighbour species, *S. perpauca* (Suppl. material 1, Genbank Accession Numbers: MH407235, MH407237, MH407239, MH407240, MH407243).

#### Remarks.

The species exhibits variation in colour patterns and several similar looking species that had been described mainly based on colour differences were synonymised with *S. flavella* by Woolley and Dal Molin (2017). It resembles *S. perpauca* (see below) but lacks the discal seta in the fore wing.

### Signiphora perpauca

Taxon classificationAnimaliaHymenopteraSigniphoridae

Girault

6AAC98FB-3A1D-5892-8EDF-71D8ED148B90

Fig. 7A–F

Signiphora perpauca Girault, 1915: 71. Female.
Signiphora woolleyi Hayat: Woolley and Dal Molin (2017).

#### Material examined.

Indonesia • 1 ♀ (on slide); West Java, Mount Halimun-Salak National Park, Sukamantri; 6.682S, 106.751E; 1007 m a.s.l.; 30-Sep-2015; MZB INDOBIOSYS-CCDB25943-H05.

#### Description (female).

**Figure 7.**
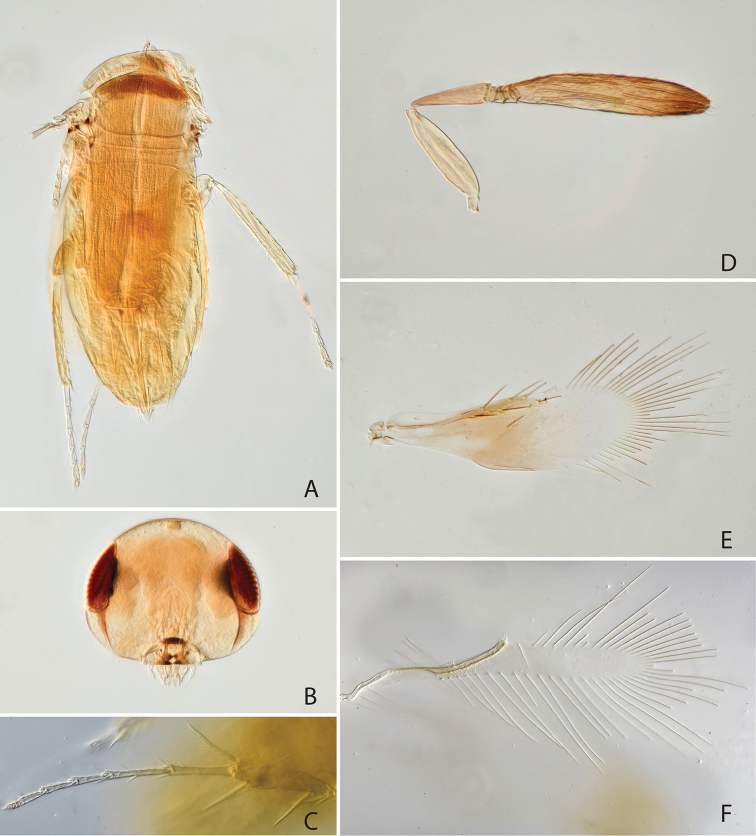
*Signiphora perpauca* Girault, female **A** body in dorsal view **B** head in frontal view **C** middle leg **D** antenna **E** fore wing **F** hind wing.

***Colour.*** Body yellow (Fig. 7A), head yellow except occiput with curved brown band along occipital carina (Fig. 7B). Antenna with scape yellow and flagellum brown, apical half of clava distinctly darker (Fig. 7D). Fore wing with dark band behind marginal vein that is proximally and distally diffusely delimited (Fig. 7E), discal seta present. Mt8 in female transverse, without a medial incision.

***Morphology.*** Head 1.76 times as broad as frontovertex width (Fig. 7B), frontovertex width 0.77 times length of clava, distance from mouth margin to facial ridge 0.57 times head height. Antenna with 3 anelli (Fig. 4D), F1 0.4 times as long as broad, F2 0.8 times as long as broad, F3 subquadrate, clava about 6 times as long as broad and 1.7 times as long as scape length (Fig. 7D), pedicel 0.77 times as long as scape length. Midlobe of mesoscutum with 2 setae on disc and anteriorly with 6 setae, scutellum with 6 setae along posterior margin. Fore wing 2.9 times as long as broad (Fig. 4E), longest setae of marginal fringe 1.4 times as long as width of disc, seta M1 present, M2b absent. Midtibial spur subequal in length to corresponding basitarsus (Fig. 4C), the latter 0.56 times as long as midtibia. Ovipositor 2.1 times as long as midtibia and 1.4 times as long as hind tibia.

#### Host.

Diaspididae (Hemiptera, Sternorrhyncha).

#### Distribution.

Widely distributed in the tropics and, apart from Indonesia, recorded from the following countries (after Woolley and Dal Molin 2017): Argentina, Cuba, Brazil, Chile, Egypt, French Polynesia, Haiti, India, Mexico, Panama, Papua New Guinea, Peru, Trinidad and Tobago, South Africa, Taiwan, Thailand, USA.

#### DNA barcode data.

The sequence of the single examined specimen is separated by 9.7% from the nearest neighbour species, *S. flavella* (Suppl. material 1, Genbank Accession Number: MH407234).

#### Remarks.

The species belongs to the *Signiphora flavopalliata* species group and can be separated from other species in the group, among other characters, by its light colour (female) and the presence of a discal seta in the fore wing (fig. 306 in Woolley and Dal Molin 2017).

### Signiphora bifasciata

Taxon classificationAnimaliaHymenopteraSigniphoridae

Ashmead

B80F23DA-CB81-5B53-A817-344112DD0E02

Signiphora bifasciata Ashmead, 1900: 411. Female.
Signiphora platensis Bréthes: Woolley (1988).

#### Diagnosis and remarks.

The Neotropical species was recorded as a parasitoid of the cycad aulacaspis scale, *Aulacaspis yasumatsui* Takagi (Hemiptera, Diaspididae) in the Bogor Botanic Gardens in 2011 (Muniappan et al. 2012). This represents the first record of the family Signiphoridae from Indonesia. The species can be separated from other *Signiphora* species by the following characters (see Muniappan et al. 2012 and figures therein): discal seta present on both fore wing and hind wing, fore wing infuscate from the wing base to the distal end of the stigmal vein, submarginal vein with 2 setae, marginal vein of the hind wing with 1 seta, female antenna with 2–3 annelli, marginal fringe of fore wing very long, nearly half as long as width of disc, mesosoma brown except scutellum and metanotum which are pale tan, yellow or white, mesoscutum with about 16 setae, scutellum with 6 setae.

## Supplementary Material

XML Treatment for Chartocerus

XML Treatment for Chartocerus javensis

XML Treatment for Chartocerus kartiniae

XML Treatment for Chartocerus sumatrensis

XML Treatment for Signiphora

XML Treatment for Signiphora bennetti

XML Treatment for Signiphora flavella

XML Treatment for Signiphora perpauca

XML Treatment for Signiphora bifasciata

## Supplementary materials

Supplementary material 1 BOLD TaxonID Tree Data type: species data https://binary.pensoft.net/file/362577This dataset is made available under the Open Database License (http://opendatacommons.org/licenses/odbl/1.0/). The Open Database License (ODbL) is a license agreement intended to allow users to freely share, modify, and use this Dataset while maintaining this same freedom for others, provided that the original source and author(s) are credited.Stefan Schmidt, Hasmiandy Hamid, Rosichon Ubaidillah, Samantha Ward, Andrew Polaszek
