# Supplementary material for: A review of the Indonesian species of the family Signiphoridae (Hymenoptera, Chalcidoidea), with description of three new species
Source: Zookeys. 2019 Dec 9;897:29–47. doi: 10.3897/zookeys.897.38148 (PMC6914708; doi:10.3897/zookeys.897.38148)
Supplement: Supplementary material 1 [file zookeys-897-029-s001.pdf]

BOLD TaxonID Tree

Title	:	Tree Result - DS-INDOSIG
Date	:	09-Jul-2019
Data Type	:	Nucleotide
Distance Model	:	Kimura 2 Parameter
Marker	:	COI-5P
Colourization	:	Barcode Cluster (BIN)

Label	:	Sample ID
Label	:	Taxon
Label	:	Country
Label	:	Province/State
Label	:	Barcode Cluster (BIN)

Sequence Count	:	12
Species count	:	5
Genus count	:	2
Family count	:	1
Unidentified	:	0

BIN Count	:	4

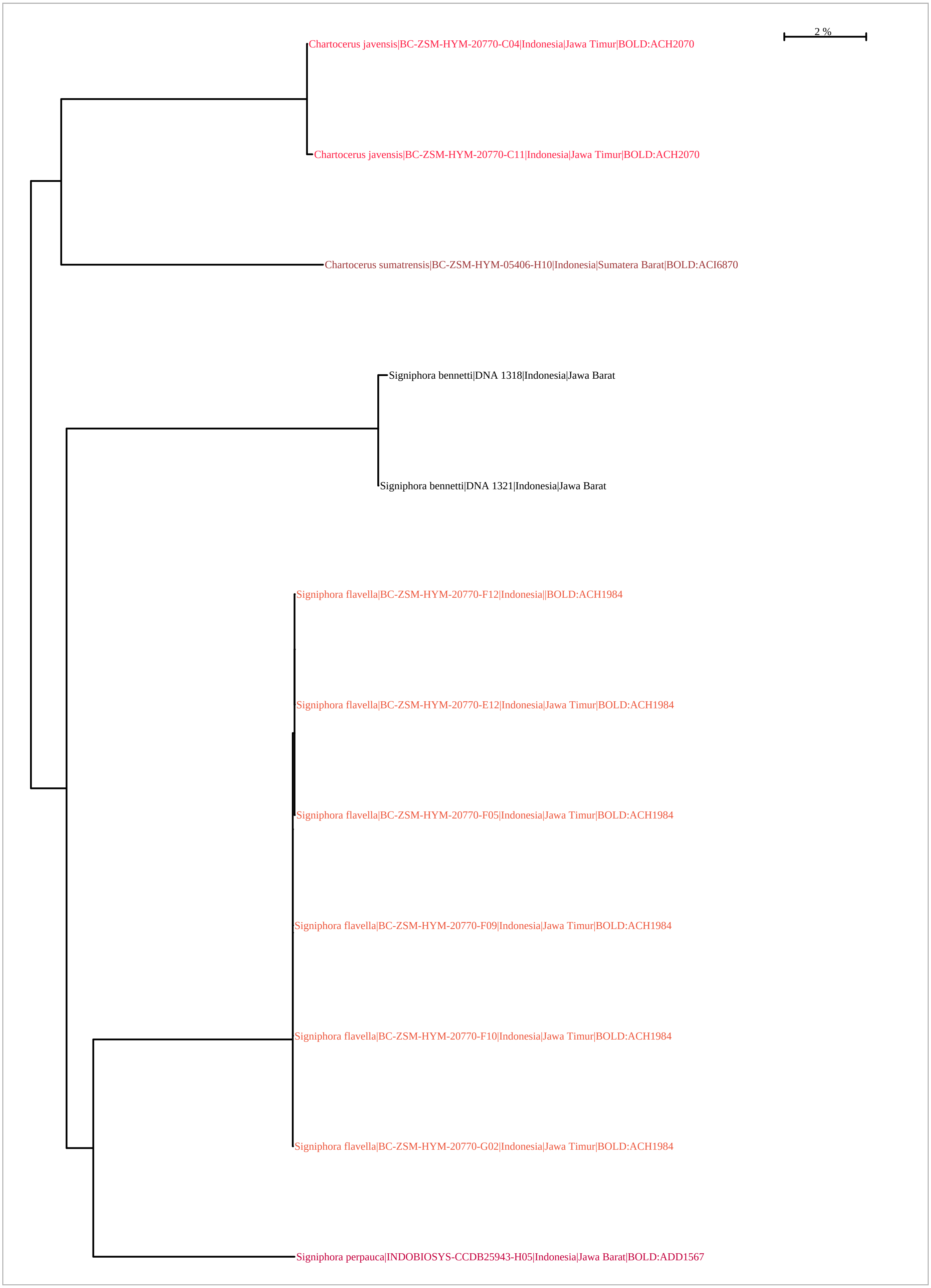
2 %
Chartocerus javensis|BC-ZSM-HYM-20770-C04|Indonesia|Jawa Timur|BOLD:ACH2070
Chartocerus javensis|BC-ZSM-HYM-20770-C11|Indonesia|Jawa Timur|BOLD:ACH2070
Chartocerus sumatrensis|BC-ZSM-HYM-05406-H10|Indonesia|Sumatera Barat|BOLD:ACI6870
Signiphora bennetti|DNA 1318|Indonesia|Jawa Barat
Signiphora bennetti|DNA 1321|Indonesia|Jawa Barat
Signiphora flavella|BC-ZSM-HYM-20770-F12|Indonesia||BOLD:ACH1984
Signiphora flavella|BC-ZSM-HYM-20770-E12|Indonesia|Jawa Timur|BOLD:ACH1984
Signiphora flavella|BC-ZSM-HYM-20770-F05|Indonesia|Jawa Timur|BOLD:ACH1984
Signiphora flavella|BC-ZSM-HYM-20770-F09|Indonesia|Jawa Timur|BOLD:ACH1984
Signiphora flavella|BC-ZSM-HYM-20770-F10|Indonesia|Jawa Timur|BOLD:ACH1984
Signiphora flavella|BC-ZSM-HYM-20770-G02|Indonesia|Jawa Timur|BOLD:ACH1984
Signiphora perpauca|INDOBIOSYS-CCDB25943-H05|Indonesia|Jawa Barat|BOLD:ADD1567
